# Similar patterns of leaf temperatures and thermal acclimation to warming in temperate and tropical tree canopies

**DOI:** 10.1093/treephys/tpad054

**Published:** 2023-04-26

**Authors:** K Y Crous, A W Cheesman, K Middleby, E I E Rogers, A Wujeska-Klause, A Y M Bouet, D S Ellsworth, M J Liddell, L A Cernusak, C V M Barton

**Affiliations:** Hawkesbury Institute for the Environment, Western Sydney University, Penrith, New South Wales 2751, Australia; Centre for Tropical Environmental and Sustainability Science (TESS) and College of Science and Engineering, James Cook University, Cairns, Queensland 4878, Australia; Centre for Tropical Environmental and Sustainability Science (TESS) and College of Science and Engineering, James Cook University, Cairns, Queensland 4878, Australia; Hawkesbury Institute for the Environment, Western Sydney University, Penrith, New South Wales 2751, Australia; Urban Studies, School of Social Science, Western Sydney University, Penrith, New South Wales 2751, Australia; Centre for Tropical Environmental and Sustainability Science (TESS) and College of Science and Engineering, James Cook University, Cairns, Queensland 4878, Australia; Hawkesbury Institute for the Environment, Western Sydney University, Penrith, New South Wales 2751, Australia; Centre for Tropical Environmental and Sustainability Science (TESS) and College of Science and Engineering, James Cook University, Cairns, Queensland 4878, Australia; Centre for Tropical Environmental and Sustainability Science (TESS) and College of Science and Engineering, James Cook University, Cairns, Queensland 4878, Australia; Hawkesbury Institute for the Environment, Western Sydney University, Penrith, New South Wales 2751, Australia

**Keywords:** *Eucalyptus*, photosynthesis, respiration

## Abstract

As the global climate warms, a key question is how increased leaf temperatures will affect tree physiology and the coupling between leaf and air temperatures in forests. To explore the impact of increasing temperatures on plant performance in open air, we warmed leaves in the canopy of two mature evergreen forests, a temperate *Eucalyptus* woodland and a tropical rainforest. The leaf heaters consistently maintained leaves at a target of 4 °C above ambient leaf temperatures. Ambient leaf temperatures (*T*_leaf_) were mostly coupled to air temperatures (*T*_air_), but at times, leaves could be 8–10 °C warmer than ambient air temperatures, especially in full sun. At both sites, *T*_leaf_ was warmer at higher air temperatures (*T*_air_ > 25 °C), but was cooler at lower *T*_air_, contrary to the ‘leaf homeothermy hypothesis’. Warmed leaves showed significantly lower stomatal conductance (−0.05 mol m^−2^ s^−1^ or −43% across species) and net photosynthesis (−3.91 μmol m^−2^ s^−1^ or −39%), with similar rates in leaf respiration rates at a common temperature (no acclimation). Increased canopy leaf temperatures due to future warming could reduce carbon assimilation via reduced photosynthesis in these forests, potentially weakening the land carbon sink in tropical and temperate forests.

## Graphical abstract

**Figure:**
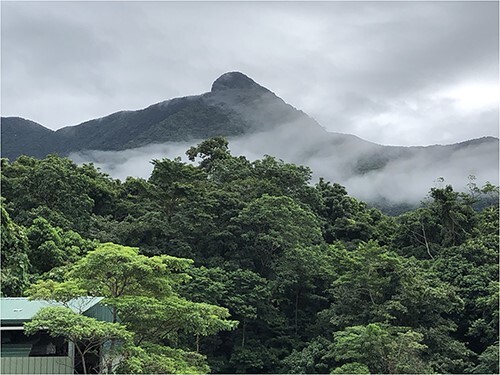

## Introduction

Average air temperatures in Australia have increased by 1.4 °C since 1910 (Bureau of Meteorology 2022) and are predicted to further increase by 2–5 °C by the end of this century (Friedlingstein et al. 2019, Masson-Delmotte et al. 2022). These predictions of increased global temperatures, though seeming small, can have profound physiological effects on plant processes such as photosynthesis and respiration (Way and Yamori 2014, Crous et al. 2022). Photosynthesis is generally increased with warming in cooler climates in part because plants frequently operate below their photosynthetic temperature optimum (Ghannoum and Way 2011, Way and Yamori 2014). By contrast, warming may have negative effects on canopy carbon uptake in ecosystems in already warm climates (Hubau et al. 2020, Sullivan et al. 2020, Crous et al. 2022). Similarly, reductions in stomatal conductance may occur together with reduced photosynthesis at warmer temperatures, especially beyond the photosynthetic temperature optimum. However, large uncertainties still remain regarding these responses to warming (Booth et al. 2012, Lombardozzi et al. 2015, Mercado et al. 2018). For instance, the inherent variability of leaf temperature in warming experiments leads to uncertainties in the evaluation of warming effects on leaf gas exchange and growth, given that leaf temperature is the main variable for many physiological processes. In order to fully evaluate the results from warming experiments, it is critical to know the effective leaf temperature achieved in these experiments, either by directly measuring leaf temperature (Slot et al. 2014, Fauset et al. 2018) or by energy balance simulations, which are rare in warming experiments (Kimball 2015).

Leaf temperature is the balance of a number of energy absorption and release processes in leaves (Campbell and Norman 1998). Both physical conditions, such as solar radiation, wind speed and air vapor pressure deficit (VPD), and biological factors, such as canopy structure, leaf size and stomatal responses, can add to variability in leaf temperature and hence may change the effective treatment size in warming experiments. Windy conditions pose a challenge to maintaining a warming treatment in the canopy, while warming on top of high ambient summer temperatures can damage leaves. Thus, it is important to conduct warming experiments in natural conditions, while measuring leaf temperatures to understand the physical and biological drivers influencing leaf temperatures and warming treatments. Results from warming experiments in intact forests are necessary to forecast the impact of future warming and underpin models to estimate the carbon balance and potential avenues for future ecosystem management (Polasky et al. 2011).

While leaf temperatures (*T*_leaf_) are generally well-coupled to air temperatures (*T*_air_) under typical environmental conditions, leaf temperatures can also warm quickly in variable environmental conditions (Leigh et al. 2012, Fauset et al. 2018, Drake et al. 2020). While not many studies have investigated the coupling between *T*_leaf_ and *T*_air_, several tropical studies have reported that *T*_leaf_ can be >10 °C warmer than surrounding *T*_air_ in sunlit leaves (Doughty and Goulden 2008, Rey-Sanchez et al. 2017) due to varying microclimate (Fauset et al. 2018) and stomatal response times (Leigh et al. 2012). By contrast, the ‘limited leaf homeothermy hypothesis’ proposed by Mahan and Upchurch (1988) posits that leaves should cool below air temperature when above ~25 °C to optimize photosynthesis but should be warmer than air temperature at lower temperatures (i.e., <25 °C) (Dong et al. 2017). This hypothesis implies that leaves actively transpire more when leaf temperatures are higher than air temperatures but is in contrast with the observed temperature extremes referenced earlier. We measured continuous leaf temperatures in the canopy for 3 months to test how well *T*_leaf_ is coupled to *T*_air_ and to evaluate the limited homeothermy hypothesis, because in certain experimental conditions, leaf warming could lead to much larger increasing *T*_leaf_ than the air temperature warming employed.

Sustained increases in *T*_leaf_ may drive adjustments in metabolic processes occurring in the leaf, called thermal acclimation (Atkin et al. 2005, Way and Yamori 2014). Physiological processes of photosynthesis and respiration are affected by leaf temperatures both in the short term (minutes to hours; Salvucci and Crafts-Brandner 2004) and the longer term (days to months) over which physiological acclimation can occur. Common acclimation responses of photosynthesis to warming involve an increase in the temperature optimum of photosynthesis and sometimes an increase in the rate of photosynthesis (Way and Yamori 2014, Crous et al. 2022). Previous work contrasting temperate and tropical species have found reduced carboxylation capacity (*V*_cmax25_, Scafaro et al. 2017, Crous et al. 2018) or reduced electron transport (*J*_max25_, Choury et al. 2022) as temperatures increased, together with reduced leaf N in species grown at higher temperatures (Xiang et al. 2013, Scafaro et al. 2017, Dusenge et al. 2021, Crous et al. 2022), resulting in lower photosynthesis rates in tropical species compared with temperate species (Xiang et al. 2013, Scafaro et al. 2017). Several studies have found reduced photosynthesis rates in tropical species with warming, especially at growth temperatures >30 °C (Cunningham and Read 2003*a*, Doughty 2011, Slot and Winter 2016, Scafaro et al. 2017, Crous et al. 2018, Carter et al. 2020, 2021, Dusenge et al. 2021). However, tropical species had higher temperature optima compared with temperate species (Cunningham and Read 2003*a*, Kumarathunge et al. 2019, Choury et al. 2022, Crous et al. 2022), while also being more heat tolerant (Cunningham and Read 2006, Slot et al. 2021), and their maximum growth rates occurred at higher temperatures compared to temperate species (Cunningham and Read 2003*a*). A recent review on evergreen trees revealed that the warming response of net photosynthesis at a common temperature depends on the average summer temperatures of a given location, with a negative response to warming in places with higher summer temperatures such as the tropics (Crous et al. 2022). This response is likely related to the limited acclimation capacity to warming in tropical species (Cunningham and Read 2003*b*, Carter et al. 2020, Dusenge et al. 2021) adjusted to a thermally stable environment compared to temperate species, which experience more temperature variability throughout the year (Janzen 1967).

In contrast to photosynthesis, leaf respiration generally responds in a universally predictable manner (Heskel et al. 2016) with similar adjustments to a temperature change regardless of whether this change was due to seasonal temperature changes or experimental warming (Aspinwall et al. 2016, Reich et al. 2016). Atkin et al. (2015) found reduced respiration rates measured at a common temperature with warmer temperatures. Leaf respiration can acclimate to higher temperatures, resulting in lower respiration rates and a lower temperature sensitivity (shallower slope of the respiration–temperature relationship) (Atkin et al. 2005). Reduced leaf respiration rates in response to warming generally improves the net carbon uptake compared with when acclimation would not have occurred. Given that the capacity of photosynthesis and respiration to acclimate to warming can vary with growth temperatures, it is important to contrast warming experiments from different latitudes and quantify which processes have acclimated.

With the predicted increases in *T*_air_ due to global warming, evaporative demand will likely increase because of rising VPD in many regions (Grossiord et al. 2020). High leaf-to-air VPD will induce stomatal closure to minimize water loss (Oren et al. 1999, Grossiord et al. 2020, Lopez et al. 2021) which, in turn, affects CO_2_ assimilation, oftentimes reducing rates in C3 plants. It is currently unclear how stomata respond to higher temperatures in combination with increased VPD, especially in mature trees (Lamba et al. 2018). Due to the non-linear response of VPD with increasing *T*_air_, warming and VPD responses are inherently linked and are hard to separate. Sustained exposures to both increased temperature and VPD are expected to reduce stomatal conductance along with a reduced intracellular to extracellular [CO_2_] i.e., *C*_i_:*C*_a_ ratio (Sulman et al. 2016, Lamba et al. 2018) in line with the least-cost theory (Prentice et al. 2014). Decreased stomatal conductance can translate into higher leaf temperatures if transpiration is also reduced. However, opposite responses have also been reported with increased stomatal conductance in response to higher temperatures. Thus, it remains unclear how to accurately represent stomatal conductance responses to leaf warming and concomitant elevated VPD. Without an accurate stomatal conductance response or any form of thermal acclimation of photosynthesis and respiration, it is highly likely that the impact of warming is currently overestimated in Earth System models (Luo et al. 2008).

Most of our current understanding of tree responses to warming is based on experimental work conducted on seedlings in controlled environments (Way and Sage 2008, Ghannoum et al. 2010, Drake et al. 2015, Sendall et al. 2015). Warming experiments on larger trees are limited by infrastructure (Luxmoore et al. 1998) despite the substantial contribution of large and mature trees to global CO_2_ uptake (Luyssaert et al. 2008). A set of warming experiments on larger trees (up to 10 m tall) have been conducted in open-top chambers (Collins et al. 2018) or whole tree chambers (Crous et al. 2013, Wallin et al. 2013, Drake et al. 2016). Only a handful of experiments have involved warming treatments in the canopy of mature trees (Nakamura et al. 2010, Doughty 2011, Slot et al. 2014, Carter and Cavaleri 2018, Carter et al. 2021). The lack of field-based warming studies in the canopy of mature forests limits our understanding of the interplay between elevated temperatures and plant performance, which in turn restricts our ability to predict the impact of climate change induced by elevated air temperatures on forests around the globe.

To fill this knowledge gap, we conducted a leaf-level warming experiment on the upper canopy leaves of mature trees in two different forest locations in Australia, a temperate *Eucalyptus* woodland and a northern tropical rainforest. Our aims were to test whether leaf warming could be achieved with sufficient precision in contrasting environments (warm-humid vs warm-dry conditions), to evaluate the coupling between *T*_leaf_ and *T*_air_ in these forests and to examine how leaf physiology responds to a similar heating magnitude for two major evergreen broadleaf forest types located in different climate zones. To test the leaf homeothermy hypothesis, we hypothesized that we would observe lower *T*_leaf_ than *T*_air_ in warmer ambient conditions and higher *T*_leaf_ than *T*_air_ in cooler conditions with a crossover temperature of around 25 °C (Dong et al. 2017). Second, to test whether we achieved consistent leaf warming in the canopy, we hypothesized that wind speed would have a negative effect on warmed *T*_leaf_, challenging the ability of the leaf heaters to maintain a consistent temperature in these conditions. Lastly, we tested the physiological impacts of warming, hypothesizing that rates of photosynthesis, stomatal conductance and respiration would all be reduced in warmed leaves compared with control leaves.

## Materials and methods

### Study location and meteorological measurements

Leaf warming was conducted at two sites in Australia with mature trees, one at the *Eucalyptus* Free-Air CO_2_ Enrichment (EucFACE) experiment in a temperate climate in southern New South Wales and one at the Daintree Rainforest Observatory (DRO) in a tropical climate in northern Queensland. The leaf warming treatments were conducted at heights >20 m, facilitated by canopy cranes at each site, which could be used to access the canopies for installation and monitoring.

The EucFACE experiment is located in western Sydney, (33°37′S, 150°44′E, 30 m a.s.l.) in a warm-temperate climate with a mean annual temperature of 17 °C and mean annual precipitation of 810 mm (1881–2022, Bureau of Meteorology, station 067105 in Richmond, NSW Australia, http://www.bom.gov.au). The open woodland (600–1000 trees ha^−1^) is dominated by *Eucalyptus tereticornis* Sm. with an upper canopy height between 21 and 24 m. For this study, only trees in ambient CO_2_ plots were accessed with a 43-m tall Jaso Crane (J4010, Idiazábal, Spain) with a 35-m jib. Air temperature, relative humidity (HUMICAP HMP 155 Vaisala, Vantaa, Finland), wind speed (Wincap Ultrasonic WMT700 Vaisala, Vantaa, Finland) and photosynthetic active radiation (PAR, LI-190, LI-COR, Inc, Lincoln, NE, USA) were monitored on a continuous basis, and 1-min averages were recorded on data loggers (CR3000, Campbell Scientific Australia, Townsville, Australia). Humidity, temperature, PAR and wind speed sensors were located on the top of the central tower of an ambient CO_2_ plot. For more information on EucFACE plots and related sensor measurements, see Gimeno et al. (2018).

The DRO in Cape Tribulation (16°06′S, 145°26′E; 50 m a.s.l.) is located in a tropical climate with a mean annual temperature of 24.4 °C and mean annual precipitation of 4586 mm (2006–2022, DRO climate station), falling mostly between December and April. The plot size is one hectare with ~85 species. The canopy was accessed with a 48-m tall Liebherr Crane (Liebherr 91, EC, Bulli, Switzerland) with 50-m jib. The forest is classified as complex mesophyll vine forest (Webb 1959) and the canopy is heterogenous with tree species with heights >30 m, such as *Castanospermum australe*A.Cunn. ex. Mudie, and co-dominant species with tree heights between 15 and 26 m, such as *Endiandra microneura*C.T.White and *Myristica globosa* subsp. *muelleri* (Warb.) W.J.de Wilde. There is also significant liana coverage in the canopy (Buckton et al. 2019). Climate data were collected using a permanently mounted automatic weather station on top of the crane platform and included measurements of rainfall, solar radiation (incoming shortwave), relative humidity wind speed and air temperature. The weather station consisted of a data-logger (CR1000, Campbell Scientific, Townsville, Australia); a weather transmitter with temperature and humidity (Vaisala HMP60, Vantaa, Finland), rainfall, wind direction and speed sensors (RMY 05103, Campbell Scientific, Townsville, Australia) and a pyranometer (SQ521, Apogee, Logan, UT, USA). A tipping bucket rain gage (RIM8000, Campbell Scientific, Townsville, Australia) was also used to record precipitation. More detailed information about the DRO can be found in Tng et al. (2016).

All climate data were averaged to 5- or 10-min intervals for consistency among sites and variables in further analyses. Atmospheric water VPD was calculated from continuous temperature and humidity measurements. Leaf-to-air vapor pressure differences (LAVPDs) were calculated in a similar way as for VPD using the appropriate leaf temperatures for each treatment instead of air temperatures.

### Leaf warming in the canopy

We selected four *E. tereticornis* trees in ambient CO_2_ conditions at the EucFACE (*n* = 8) Cumberland Plain Forest and three *M. globosa* ssp. *muelleri* trees at the DRO (*n* = 6). Both of these species are dominant or co-dominant in the forest on each site. In each of the selected trees, two leaf heater pairs (one reference leaf that was unheated and one heated leaf in a paired design; see Supplemental Methods SM1 available as Supplementary data at *Tree Physiology* Online) were installed at a canopy height of ~20–22 m between November 2019 and February 2020 at EucFACE and between May and July 2021 at the DRO.

The leaf heaters utilized a unique design (see Supplemental Methods SM1 available as Supplementary data at *Tree Physiology* Online) with proportional heating through a silicon-coated nichrome heating wire with 15 W of heating capacity rather than controlling temperature in an on/off mode. They consisted of a clear, plastic box containing holes for air circulation and fishing wire to hold the leaf in place at a constant distance from the heating wire (Supplemental Methods SM1 and Figure S1 available as Supplementary data at *Tree Physiology* Online). The boxes were held in place by small, custom-built metal frames attached to a large branch via cable ties. The spacing of paired reference leaf box (unheated) and the warmed leaf box was typically ~1 m, which was installed with similar aspect and light conditions. All leaf heaters were installed at branch tips in the upper canopy.

### Relative and absolute leaf temperatures

The difference in temperature between a heated leaf and its reference leaf was measured with a pair of 30-gage (~0.25 mm diameter) copper-constantan type-T thermocouples (Omega Engineering Inc, Norwalk, CT, USA) connected via a common constantan wire. The thermocouples were installed on the underside of both the reference and heated leaves such that they were touching at all times. Surgical tape (Micropore 3M, St. Paul, MN, USA) was used to keep the thermocouples in place and this was followed up by monthly visual check-ups (Figure S1 available as Supplementary data at *Tree Physiology* Online). Thermocouple wires were then connected to an AM25T multiplexer (Campbell Scientific, Townsville, Australia) and a CR1000 Campbell datalogger (Campbell Scientific, Townsville, Australia). Additional 36-gage (0.13 mm diameter) copper-constantan thermocouples (Omega Engineering Inc., Norwalk, CT, USA)) were installed on two reference leaves to measure the absolute leaf temperatures at each site.

Temperature control for the heaters was achieved by monitoring the temperature difference between heated and reference leaves (*T*_diff_) and adjusting the power to the heating wire to maintain the desired differential of ~4 °C (Supplemental Methods available as Supplementary data at *Tree Physiology* Online). Power was regulated by a custom-made electronic circuit board installed to accommodate eight leaf heater pairs. The logger measured the temperature differences every 5 s, and the power to each heater was updated every 30 s with a PID algorithm. The leaf heater program was optimized to avoid temperature spikes and to achieve optimal heating between 3.5 and 4.5 °C above the reference leaf.

To test whether the box infrastructure affected actual leaf temperatures, we used an infrared thermal camera (FLIR T1010 28 °C thermal imaging camera, Teledyne FLIR LLC, OR, USA) to take photos of both the reference and surrounding leaves in the canopy. Leaf temperatures of the reference leaves in the boxes were not different from the surrounding leaves (Figure S2 available as Supplementary data at *Tree Physiology* Online). Leaf temperatures were recorded between November and December 2019 at EucFACE and between May and July 2021 at DRO to test how well the leaf temperatures in the reference leaves were coupled to surrounding air temperatures. The difference between leaf temperature and air temperatures is indicated as Δ*T* (i.e., *T*_leaf_ − *T*_air_).

### Gas exchange measurements

Gas exchange measurements were conducted to test whether the warming experiment induced changes in leaf physiology. At EucFACE, several extreme temperature events occurred during the warming treatment throughout the Australian summer of 2019–20, including a 3-day heatwave at the end of January 2020, where air temperatures exceeded 41 °C. The extreme temperatures caused leaf browning, particularly in the heated leaves, which reached leaf temperatures of ~50 °C at times during this record-breaking summer. Consequently, only three intact leaf heater pairs where both the reference and warmed leaf had remained green were left to measure for gas exchange. Therefore, a replication of three *E. tereticornis* leaves (from three individual trees) was used for gas exchange purposes.

Gas exchange measurements of photosynthesis under saturating light conditions of 1800 μmol m^−2^ s^−1^ (*A*_net_) and light- and CO_2_-saturating (1800 μmol m^−2^ s^−1^ and 1800 μmol mol^−1^) conditions (*A*_max_), stomatal conductance (*g*_s_) and dark respiration (*R*_d_) were measured using an LI-6400XT with the 2 × 3 cm^2^ red-blue lamp (LI-COR Inc.) in the upper canopy at a constant leaf temperature (25 °C at EucFACE and 30 °C at DRO) on three leaf heater pairs (*n* = 3). Saturating light and CO_2_ conditions were determined using light and CO_2_ response curves, respectively, measured in previous years on similar or the same species. While *A*_max_ represents a measure for photosynthetic capacity, underlying components of photosynthesis, the maximum carboxylation rate (*V*_cmax_) and the maximum electron transport rate (*J*_max_) were derived from *A*_net_–*C*_i_ curves at EucFACE only following the same protocol as Wujeska-Klause et al. (2019). Heated and non-heated reference leaves were compared at a standard temperature to assess the degree of acclimation after several weeks of exposure to +4 °C warming. After these measurements, small branchlets were collected in a bucket and were immediately re-cut under water. In most cases, the same leaves (otherwise the adjacent leaf) on which photosynthesis was measured were used in a respiration temperature response curve within a few hours of collection. Respiration temperature response curves were measured between 15 and 45 °C on all replicate leaves, increasing the measurement temperatures by 1 °C per min in a 10 × 10 cm^2^ gas exchange chamber (3010-GWK1, Walz, Effeltrich, Germany) connected to an infra-red gas analyzer (Licor6400XT, LI-COR Inc.) in the lab.

### Statistical analyses

All analyses and graphs were conducted in R 4.2.2. (R Core Develeopment Team 2022). Meteorological data were used to calculate the daily mean and 1 SD for air temperature, relative humidity, VPD, wind speed and PAR. Absolute leaf temperatures and the difference between sample and reference leaves (*T*_diff_) were calculated based on 5-min data from which probability density functions were derived over a period of several weeks. Linear regression was used to assess the relationships between variables. A paired *t*-test within species was used to analyze the gas exchange differences in net photosynthesis, stomatal conductance, maximum photosynthesis and dark respiration rates between reference and warmed leaves. A–C_*i*_ curves were fitted using the R ‘ecophys’ package (Duursma 2015). Temperature response curves of dark respiration were analyzed for treatment differences using the R ‘nlstools’ (Baty et al. 2015) and ‘nlshelper’ packages (Duursma 2017). A *P*-value <0.1 was considered to be statistically significant for gas exchange due to the low number of replicates (*n* = 3).

**Figure 1:**
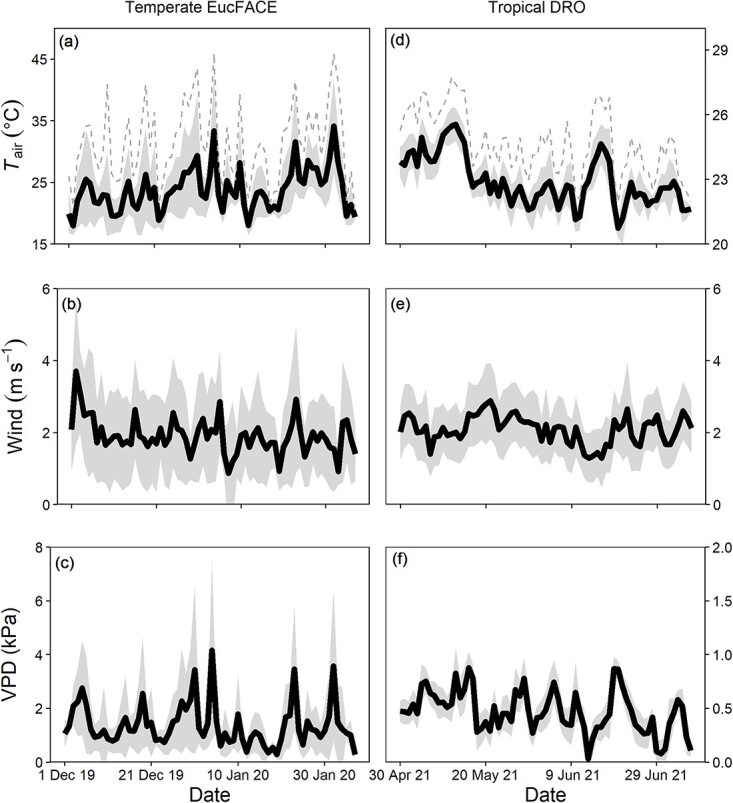
Daily means (solid line) and 1 SD (zone around line) during the experimental period at EucFACE (left panels) and at DRO (right panels) for air temperature (*T*_air_, panels a and b; note differences in scale), wind speed (panels c and d) and VPD (panels e and f; note differences in scale). The warming experiment at EucFACE was conducted over the summer of 2019–20, while the experiment at DRO covered from May to August 2021. The dashed lines in panels a and d indicate the daily maximum air temperatures across the study period.

## Results

### Background environmental conditions for leaf warming

The efficacy and outcome of warming experiments can depend on the background environmental conditions under which they are conducted (Aronson and McNulty 2009). For the *Eucalyptus* leaf warming experiment at EucFACE, the prevailing conditions were hot and windy, with several extreme temperature events and extensive bushfires occurring at that time in Australia (Boer et al. 2020). On average, across the study period, air temperatures were 23.8 °C, ranging between 11 and 46 °C. Over the course of the first two summer months (December–January 2019–20 in Australia), there were 9 days of air temperature >40 °C, with two extreme temperature days (Figure 1a). The first extreme heat event (4 January 2020) lasted for 6.5 h with ambient temperatures >44 °C, which resulted in the loss of one heated leaf (out of the eight leaf pairs installed). However, five more leaves were lost during a 3-day heatwave at the end of January 2020 (31 January–2 February), where temperatures exceeded 41 °C on each day with that on 1 February exceeding 45 °C, resulting in only three intact leaf heater pairs left for gas exchange measurements. Wind speed was on average 1.9 m s^−1^ (Figure 1b), but high wind gusts >6 m s^−1^ (based on 5-min averages) occurred on 21 days during the 47-day experiment. Along with high temperatures (>39 °C), high VPDs occurred with peaks >6 kPa on 8 days and an average of 1.4 kPa over the experimental period (Figure 1c). The fourth of January 2020 set a record maximum temperature at EucFACE of 47.1 °C along with a record high VPD of 9.5 kPa. The site was exceptionally dry with soil moisture content in the top 3 m in early 2020 being the lowest recorded in 8 years at EucFACE (in the 5th percentile of all observations, Figures S3 available as Supplementary data at *Tree Physiology* Online).

Environmental conditions during the dry season at the DRO were less extreme (Figure 1, right panels). Commensurate with the rainforest location, the average air temperature was 22.9 °C between the periods of May and July (Figure 1b), with an average relative humidity of 83% and an average VPD of 0.47 kPa (Figure 1f). Wind speed was on average 2 m s^−1^, ranging between 0 and 6.5 m s^−1^, based on 5-min averages. At DRO, six leaves (three pairs) were installed in *M. globosa*, and none was lost at the time of this experiment.

### Leaf warming at two sites

**Figure 2:**
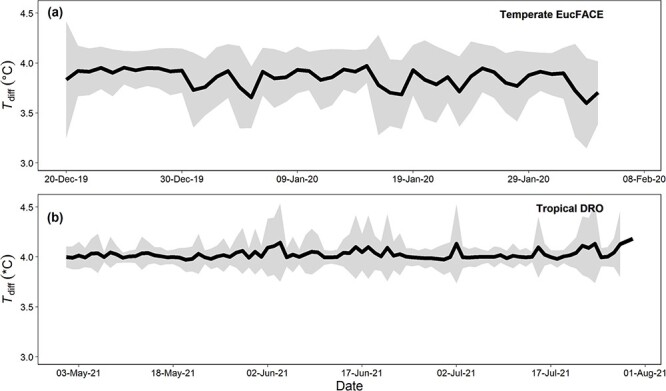
Overall leaf heater performance indicated by temperature differential (*T*_diff_) between reference and warmed leaf pairs across six to eight leaf heaters at EucFACE (panel a) and at the DRO (panel b). The target was to warm 4 °C above the temperature of the reference leaf (*T*_diff_ = 4), which was maintained in both experiments across several months in the field. Daily means are the indicated as a solid line, with the area around the line representing 1 SD of the mean.

**Figure 3:**
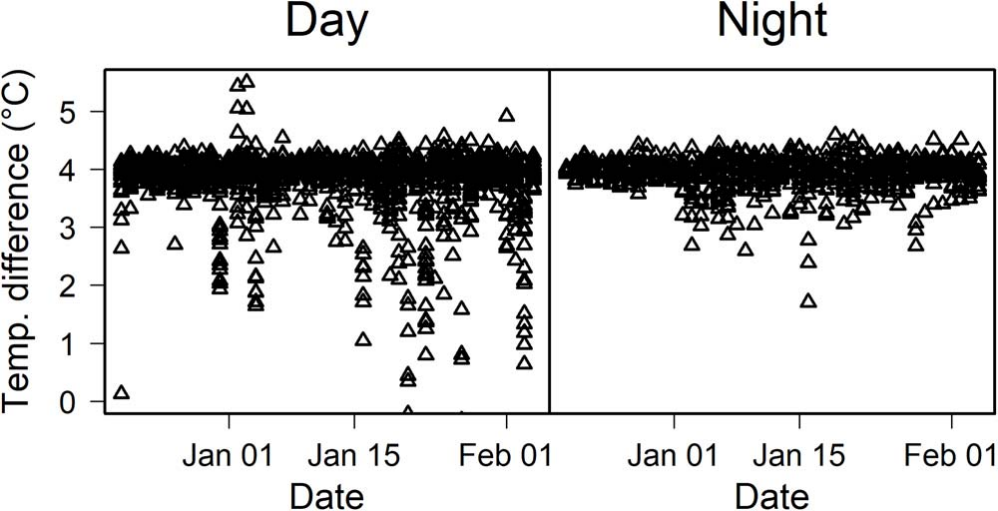
Example of a day–night contrast in leaf heating performance with a target of 4 °C above the reference leaf at EucFACE, indicated at ‘temperature difference’ (*T*_diff_). While the target is met both in day and night, night-time conditions are generally more stable due to lack of radiation and winds.

We evaluated the ability of our heating design to maintain *T*_leaf_ above reference leaf temperatures under a set of challenging conditions: extreme heat with high wind speeds (EucFACE) and high radiation combined with high rainfall (DRO). Leaf heating at EucFACE was maintained at +3.9 ± 0.3 °C (mean across all leaves over time ± 1 SD) above the temperature of the reference leaves (Figure 2a). Seven out of eight heating sensors had good heating performance, while one defective sensor gave intermittent values due to a bad connection and was omitted from further analysis. Across 47 days of experimental warming at EucFACE and 68 days at DRO, leaf heaters did not always work at full capacity, as measured via the duty cycle. The duty cycle was 63 ± 18% on average at EucFACE and was 39 ± 9% at the DRO, indicating that the full 15 W (or 100%) was not always needed to achieve the target warming of +4 °C above reference leaf temperatures. At the DRO, the leaf heating achieved +4.0 ± 0.1 °C above the reference leaf temperature (Figure 2b). At night with low wind and no radiation load, the warming versus reference leaf temperature difference was even more stable compared with during the day (Figure 3). Thus, the paired leaf heating produced a consistent warming signal at both sites and performed very close to the target of 4 °C continuous warming in spite of large contrasts in environmental conditions at the two sites (Figure 1).

**Figure 4:**
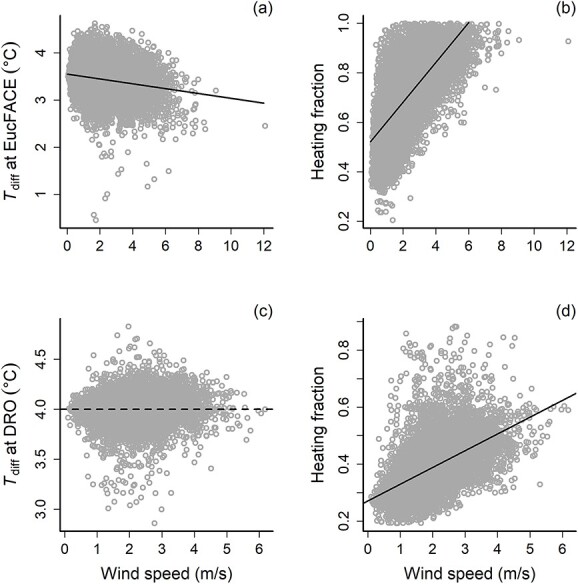
Relationships between leaf heater performance (target temperature difference between warmed and reference leaves, *T*_diff_, left panels) and heating fraction (i.e., the duty cycle or the fraction of heat applied to reach the target temperature, right panels) as a function of wind speed at the EucFACE site (panels a and b) and the DRO site (panels c and d). While there was no relationship between leaf heater performance and wind speed at the DRO, there was a slight negative relationship at EucFACE (*T*_diff_ = −0.051 * wind speed + 3.55; *R*^2^ = 0.04, *P* < 0.0001) due to the higher wind speeds experienced. The heating feedback system rapidly increased the heating fraction in response to increased wind speed, counteracting the heat loss, with a steep increase in heating fraction to higher winds (up to 6 m s^−1^) at both sites.

A priori, we expected that wind speed would affect the efficacy of leaf heating because wind can be variable and gusty at times. At EucFACE, there was a significant but weak negative relationship between the target leaf temperature differential (*T*_diff_) and wind speed (*R*^2^ = 0.16, *P* < 0.0001), but the slope of that relationship was small (−0.096, Figure 4a), indicating that leaf heating was only slightly affected by wind speed. The duty cycle (i.e., fraction of maximum heating power) increased with wind speed, indicating the need for more power required to achieve the target temperature differential of 4 °C warming (Figure 4b). There was no relationship between *T*_diff_ and wind speed at the tropical site, DRO (Figure 4c), but peak and mean wind speeds were lower compared with EucFACE. With the smaller range of wind speeds at the DRO, the duty cycle of warming linearly increased with wind speed (*R*^2^ = 0.28, *P* < 0.0001, Figure 4d). In short, the heating system successfully achieved what it was designed for, to increase heat output (i.e., duty cycle or heating fraction) in response to increased convective heat loss at higher wind speeds.

Our experimental set-up reflected natural conditions expected in the future by combining increased temperatures and increased VPD. When *T*_leaf_ was higher than *T*_air_, LAVPD was higher than VPD, whereas when *T*_leaf_ was lower than *T*_air_, the LAVPD was lower than the VPD. This means that the differences between *T*_leaf_ and *T*_air_ were largest at the more extreme temperatures (warmer or cooler) across the measured range but converged in the middle of the range. There was an exponential increase in LAVPD as a function of *T*_air_ with an overall higher LAVPD in warmed leaves than in ambient leaves (Figure S4 available as Supplementary data at *Tree Physiology* Online). At both sites, there was a shift toward higher LAVPD with warming due to increased VPD in warmed leaves (Figure S4 available as Supplementary data at *Tree Physiology* Online). At moderate temperatures near 25 °C, the LAVPD shift in warmed leaves was on average 0.71 kPa at EUCFACE and was 0.91 kPa at DRO compared with non-warmed reference leaves.

### Coupling of leaf temperatures with air temperatures in reference leaves

**Figure 5:**
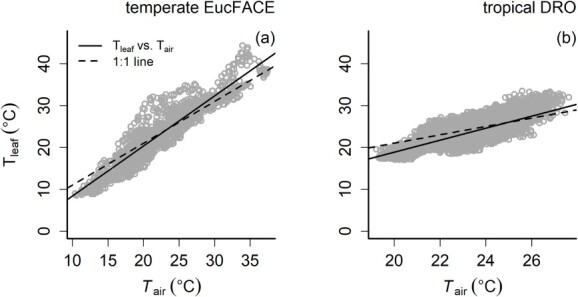
Relationship between 10-min averages of ambient (reference) leaf temperatures (*T*_leaf_) and air temperatures (*T*_air_) in *E. tereticornis* (panel a, 2252 observations from four healthy leaves) and *M. globosa* (panel b, 9816 observations from three healthy leaves) over a period of up to 3 months. The *T*_leaf_ and *T*_air_ were positively related and close to the 1:1 line at both sites, with a larger air temperature range experienced at EucFACE compared with tropical DRO. At EucFACE, the relationship was *T*_leaf_ = 1.2 * *T*_air_ − 3.6; *R*^2^ = 0.89, *P* < 0.0001 (panel a); and at the DRO, the relationship was *T*_leaf_ = 1.4 * *T*_air_ − 9.8; *R*^2^ = 0.66, *P* < 0.0001 (panel b). At warmer air temperatures (> 25 °C), leaf temperatures were warmer than air temperature, with a crossover point around 25 °C below which leaf temperatures were cooler than air temperatures.

**Figure 6:**
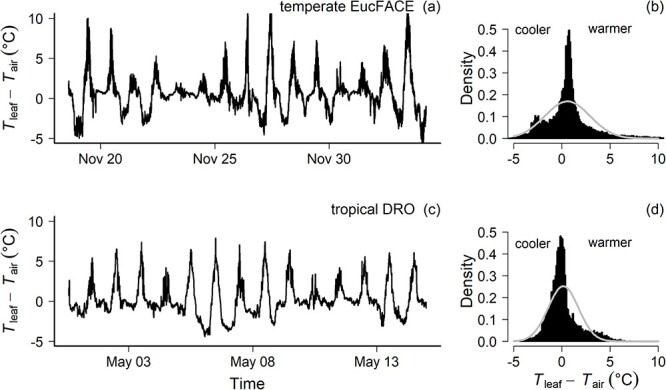
Difference between ambient (non-heated) leaf temperatures (*T*_leaf_) and air temperatures (*T*_air_) across 5 weeks (left panels) for EucFACE (panels a and b) and DRO (panels c and d) for the three to four healthy reference leaves. Leaf temperatures were up to 8–10 °C warmer than air temperatures at times in the day. A probability density function at each site (panels b and d), overlayed with a normal distribution represented by the solid line, indicates the average leaf temperatures experienced over 24 h (close to zero, but slightly positive) while also showing that leaves were warmer than air temperature more of the time across both study periods.

At EucFACE, reference leaf temperatures (e.g., non-warmed control) were well coupled to air temperatures based on the strong correlation between *T*_leaf_ and *T*_air_ (*R*^2^ = 0.89, Figure 5a), although the leaf–air temperature relationship was not entirely one-to-one, with a slope of 1.2. Leaf temperatures at EucFACE ranged between 10 and 38 °C between November and December 2019. Leaf temperatures tended to be warmer than air temperatures >23 °C and cooler than air temperatures <23 °C (crossover point where *T*_leaf_ = *T*_air_, Fig. 5). At EucFACE, temperature differences between reference leaf and air temperatures (Δ*T* = *T*_leaf_ − *T*_air_) ranged from the minimum Δ*T* of −5.5 °C (often at night) to the maximum Δ*T* of +12.06 °C when leaves were sunlit (Figure 6a). On average across 24 h, the diel Δ*T* was 0.58 °C, with leaves being somewhat warmer compared with air temperatures for 60% of the time (Figure 6b).

Leaf temperatures at DRO ranged between 18 and 28 °C between May and July 2021. The *T*_leaf_−*T*_air_ relationship at DRO was somewhat more variable than at EucFACE, though with a similar slope of 1.4 (*R*^2^ = 0.66, vs *R*^2^ = 0.89 at EucFACE). The crossover point between leaves being warmer or cooler than air temperature was around 25 °C for this site (Figure 5b). The diel temperature differences between leaf and air temperatures ranged from the minimum Δ*T* of  −4.4 °C up to the maximum Δ*T* of +7.9 °C relative to air temperature (Figure 6c) with a diel average of 0.15 °C. This temperature range was similar to the temperature differences observed at EucFACE, and leaves were warmer than air temperatures for ~54% of the time (Figure 6d), again similar to leaves at EucFACE.

### Physiological responses to warming

**Figure 7:**
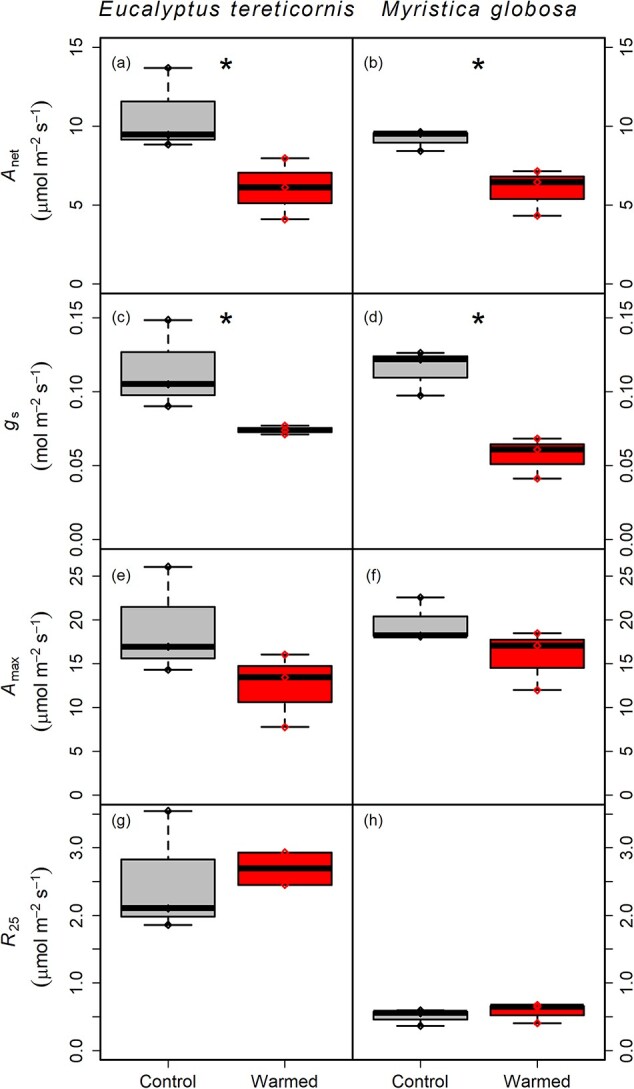
Gas exchange measured in situ at the canopy level in control (gray) and warmed (red) leaves of tree species at each site: *E. tereticornis* at EucFACE (*n* = 3, left panels) measured at 25 °C and *M. globosa* at DRO (*n* = 3, right panels) measured at 30 °C except for *R*_25_. Data shown are for net photosynthesis (*A*_net_, panels a and b), stomatal conductance (*g*_s_, panels c and d) and CO_2_- and light-saturated photosynthesis (*A*_max_, panels e and f) and mitochondrial respiration measured at a common temperature of 25 °C (*R*_25_, panels g and h). Stars indicate a significant difference between treatments at *P* < 0.1, with three replicates for each treatment indicated by diamond points on each panel.

To test the hypothesized effects of experimental leaf warming of 4 °C on leaf physiology, we focused on *A*_net_ and *g*_s_ at current growth temperatures and dark respiration at a common temperature of 25 °C at both sites. As hypothesized, warming had a significant and negative effect on both *A*_net_ and *g*_s_ in both species with similar effect sizes at each site. *Eucalyptus tereticornis* reduced *A*_net_ from 10.7 ± 1.5 to 6.1 ± 1.1 μmol m^−2^ s^−1^ (−43%, *P* = 0.07, Figure 7a), while *M. globosa* reduced *A*_net_ from 9.2 ± 0.4 to 6.0 ± 0.8 μmol m^−2^ s^−1^ (−35%, *P* = 0.03, Figure 7b). Stomatal conductance was reduced in response to warming, from 0.11 ± 0.02 to 0.07 ± 0.002 mol m^−2^ s^−1^ in *E. tereticornis* (−35%, *P* = 0.082, Figure 7c) and from 0.12 ± 0.01 to 0.06 ± 0.01 mol m^−2^ s^−1^ in *M. globosa* (−51%, *P* = 0.008, Figure 7d). The maximum light and CO_2_-saturated photosynthesis, *A*_max_, a measure of photosynthetic capacity, was reduced from 19.35 ± 1.73 to 14.11 ± 1.60 μmol m^−2^ s^−1^ across both species but was not significantly different between warming treatments in either species (Figure 7). The reduction in *A*_net_ was supported by the underlying biochemistry of photosynthesis, which was down-regulated in *E. tereticornis* under warming, with *V*_cmax_ and *J*_max_ being 46 and 36% reduced, respectively, in warmed leaves compared with non-warmed leaves (*P* = 0.085, Table 1).

**Figure 8:**
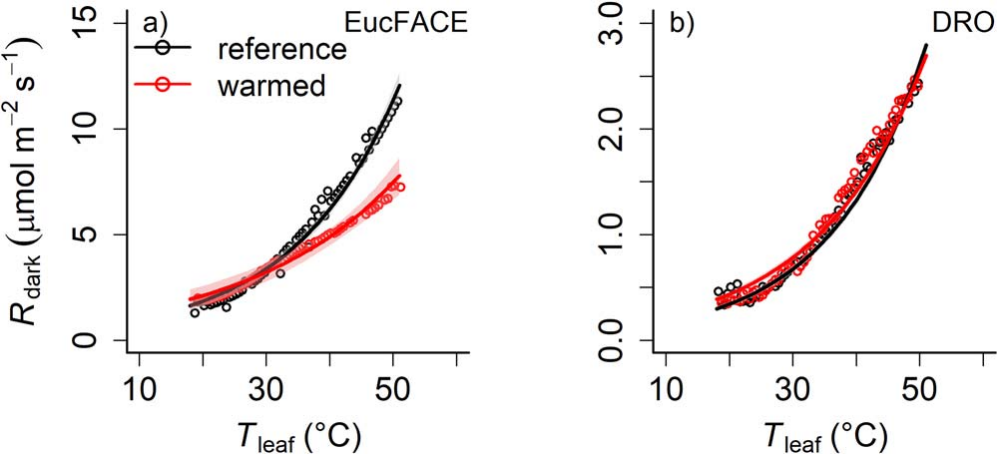
Respiration–temperature response curves for three reference (gray) and warmed (red) leaves in *E. tereticornis* at EucFACE (panel a), where the temperature sensitivity in warmed leaves was reduced compared with non-warmed reference leaves. There was no difference in the temperature-response curve of *M. globosa* (panel b).

In contrast to our expectations, respiration at 25 °C was similar in warmed versus non-warmed leaves at both sites, although the absolute respiration rates across treatments in *M. globosa* (0.54 ± 0.05 μmol m^−2^ s^−1^) were much lower compared with *E. tereticornis* (2.55 ± 0.05 μmol m^−2^ s^−1^Figure 7g and h). There was a shallower slope (i.e., lower *Q*_10_) in the respiration–temperature response curve in warmed leaves of *E. tereticornis*, with a *Q*_10_ of 1.52 ± 0.02 compared with 1.83 ± 0.05 in the control leaves (Figure 8), a reduction of 17% (*P* < 0.001). The temperature response curves of *M. globosa* in warmed and non-warmed leaves fell on top of each other across the whole temperature range (Figure 8b) with no difference in respiration rates or *Q*_10_ (2.36 ± 0.04) between warming treatments.

## Discussion

### Leaf warming in the canopy of mature trees

Leaf warming experiments in the canopy of tall, mature trees are rarely done due to the methodological challenges and varying environmental conditions at such heights. The majority of warming experiments are conducted in structurally simple vegetation such as grasslands or with small-stature plants (Rich et al. 2015, Wang et al. 2019). Only a few tall forest experiments have been conducted in the past (Doughty 2011, Slot et al. 2014, Carter et al. 2021) in which the photosynthesis or respiration responses to warming were evaluated. Despite varying environmental conditions at both the tropical and temperate field sites, our experimental design of leaf warming in the upper canopy consistently heated leaves 4 °C warmer and resulted in a different phenotypic response when compared with non-warmed reference leaves. Previous in situ warming experiments have used a binary warming approach (alternating heater on–heater off), which can result in large, short-term temperature fluctuations in leaf temperature (Doughty 2011, Slot et al. 2014, Carter et al. 2021). Too many temperature spikes may limit plant acclimation due to an inconsistent warming signal, resulting in an unclear warming response in the long term (months). By contrast, our approach modulated the amount of warming needed, depending on varying environmental conditions, creating a robust and stable leaf warming treatment (Figure 2). In addition, the distance between the heating wires and the leaf was consistent across all species and individuals, avoiding inconsistent results due to leaves moving away from the heater in the wind or browning due to close contact with the heating wire. Thus, several of the challenges in warming leaves in situ in the canopy of mature trees have been improved with our approach compared with previous leaf heating experiments.

Understanding how plant species will respond to warming provides crucial underpinnings for models which estimate the strength of the future terrestrial carbon sink with climate change. While controlled environments have been helpful to reveal physiological responses to warming, there is still a lack of evidence on how mature trees adjust their performance in response to changing environmental conditions in the field. This includes the interrelationships between leaf temperatures and canopy structure, plant performance and how a changing environment throughout the day (or year) affects leaf temperatures and carbon assimilation (Asner et al. 2016, Ordway and Asner 2020). Carbon uptake and plant productivity are modeled based on mechanistic leaf-level processes, so quantifying how warming affects leaves and the processes therein is critical, including the magnitude of thermal acclimation (Smith and Dukes 2013, Mercado et al. 2018). Moreover, understanding how *T*_leaf_ affects carbon uptake and plant productivity in fluctuating environmental conditions in a realistic context contributes to models that can scale-up these results and thus advance our capacity to predict future climate change effects on forests.

### Leaf temperatures in the upper canopy

Leaf temperatures are crucial to evaluating plant function in response to the environment, but within-canopy leaf temperatures which are continuously measured via thermocouples are rare. In the forest canopy, microclimate is a key driver of leaf temperatures (Campbell and Norman 1998). Understanding how temperatures are changing in a forest, including within-canopy air and leaf temperature variation, is important to predict forest responses to dynamic environmental drivers (De Frenne and Verheyen 2016, Still et al. 2022). The combination of heat loss from the leaf and transpiration represent the sensible and latent heat fluxes, which must be balanced with the net radiation at the leaf surface (Campbell and Norman 1998). Due to leaf structure, low thermal mass and the magnitude of the various resistances involved, the difference between *T*_leaf_ and *T*_air_, Δ*T*, adjusts rapidly in response to varying environmental conditions. Incoming solar radiation is an important factor to determine Δ*T* (Linacre 1964, Fauset et al. 2018). Therefore, leaf temperatures can differ by several degrees from air temperatures (Campbell and Norman 1998), and at times, by >10 °C (Gates 1965, Rey-Sanchez et al. 2017, Fauset et al. 2018, Miller et al. 2021). In agreement with this, we found leaf–air temperature differences up to 10 °C at both temperate and tropical sites (Figure 6).

Our results demonstrate that *T*_leaf_ was warmer than *T*_air_ at temperatures >25 °C at both sites. On average, for both sites, the leaf to air temperature difference was close to zero, but positive, indicating that leaves were overall somewhat warmer than the ambient air temperature (Figure 6). According to the limited leaf homeothermy hypothesis, leaves are expected to be cooler than *T*_air_ at higher temperatures due to transpiration cooling in order to maintain *T*_leaf_ within optimal temperatures for photosynthesis (Linacre 1964, Mahan and Upchurch 1988). However, it should be noted that transpiration can cool leaves at any temperature. Thus, leaves warmer than air temperatures can still be cooler than they would otherwise have been without transpiration. These results do not support the limited homeothermy hypothesis, because in forest environments, most of the incoming radiation is absorbed by leaves. Diurnally rising air temperatures measured in the canopy are a result of sensible heat transfer from leaves to air because leaves are warmer than their environment. Several field experiments have also reported no evidence of homeothermy or thermoregulation in forest canopies (Drake et al. 2020, Still et al. 2022), indicating that leaves do not cool below air temperature above a given temperature threshold. The *T*_leaf_ are often several degrees higher than *T*_air_ in upper canopy environments (Figure 5, Gates 1965, Rey-Sanchez et al. 2017, Fauset et al. 2018, Miller et al. 2021), suggesting limited thermoregulation capacity in the upper canopy (Miller et al. 2021). In natural forest settings, homeothermy is unlikely to occur, in contrast to more controlled environments with abundant water supply (Still et al. 2022). Moreover, Still et al. (2022) found that most carbon assimilation occurred when canopy *T*_leaf_ values were modestly higher than *T*_air_ across forest types.

**Table 1:** Average rates of maximum carboxylation (*V*_cmax_) and maximum electron transport (*J*_max_) in μmol m^−2^ s^−1^ across three warmed and three reference leaves derived from *A*–*C*_i_ curves in *E. tereticornis* using light-saturating conditions of 1800 mmol m^−2^ s^−1^ and an average leaf temperature of 24.92 ± 0.13 °C across all curves; *A*–*C*_i_ curves were not measured for *Myristica* during the study period.

Species	Treatment	*V*_cmax_	*J*_max_
*E. tereticornis*	Reference	57.01 ± 9.73	91.85 ± 18.71
	Warmed	31.04 ± 6.29	58.73 ± 10.87

We observed a switch where diel *T*_leaf_ went from warmer to cooler than *T*_air_ (at Δ*T* = 0 (Dong et al. 2017), varying between 23 and 25 °C (Figure 5) despite the different latitudes and climates of the two study sites (difference in mean annual temperature of 7 °C). While it has been posited that this transition tends to occur close to the temperature optimum of photosynthesis (Michaletz et al. 2016), there are clear differences in the temperature optimum of photosynthesis between temperate and tropical regions with a higher temperature optimum in the tropics (Crous et al. 2022). Moreover, the temperature optimum of photosynthesis varies seasonally and is only partly determined by previously experienced air temperatures (Crous et al. 2013). Other factors contributing to the temperature optimum of photosynthesis are the enzyme activity of underlying biochemistry (Kumarathunge et al. 2019, Choury et al. 2022), the degree of stomatal opening with temperature (Lin et al. 2012, Slot and Winter 2016) and leaf nitrogen investment into the photosynthetic apparatus (Onoda et al. 2005, Hikosaka et al. 2006), each of which can be directly or indirectly influenced by air temperature. Thus, the temperature optima of photosynthesis tend to be higher than the temperature, where Δ*T* = 0, as photosynthesis is optimized to daytime temperatures.

While leaf warming resulted in higher LAVPD, higher LAVPD also occurred in reference leaves when *T*_leaf_ > *T*_air_ (Figure S4 available as Supplementary data at *Tree Physiology* Online). Higher temperatures can affect VPD due to the exponential relationship between saturation vapor pressure and air temperatures. Thus, at high air temperatures, the VPD may have a larger effect on particular physiological processes than the warming effect itself (Figure S4 available as Supplementary data at *Tree Physiology* Online). However, the combination of increased VPD and warming represents a realistic natural scenario in line with expectations of our future climate (Masson-Delmotte et al. 2022). The exponential relationship between LAVPD and *T*_air_ was much steeper at EucFACE compared with DRO due to their different climates. The range of both *T*_air_ and LAVPD was larger at EucFACE, which is located in a much drier climate compared with DRO (annual precipitation of 810 mm vs 4586 mm for these respective sites; see also Figure S4 available as Supplementary data at *Tree Physiology* Online). However, leaf thermoregulation was similar between both sites (Figure 5), with *T*_leaf_ > *T*_air_ at temperatures >~25 °C.

### Physiological responses of photosynthesis and respiration to warming

Leaf temperature is a key variable determining the rates of respiration, photosynthesis and transpiration. In this study, we found warmer *T*_leaf_ had large effects on leaf physiology in dominant canopy species in both forest types. Reduced stomatal conductance has been predicted to be a major factor in photosynthetic decline, especially at higher temperatures. Photosynthesis and stomatal conductance were both significantly reduced (~40%) with long-term warming in both *E. tereticornis* and *M. globosa*, with caveats associated with the limited sample sizes due to logistical constraints and some leaf mortality. While there are inconsistent reports of stomatal conductance responses to warming (Marchin et al. 2016), studies on larger trees have mostly reported decreased stomatal conductance (Lamba et al. 2018, Fauset et al. 2019, Dusenge et al. 2021) with higher temperatures. Stomatal closure is a typical response to increased VPD (Grossiord et al. 2020), while reduced photosynthesis can be the result of both increased temperatures and stomatal closure due to increased VPD. The LAVPD was increased in warmed leaves (Figure S4 available as Supplementary data at *Tree Physiology* Online), suggesting that LAVPD likely had a stronger impact on *g*_s_ than a temperature increase leading to stomatal closure in warmed leaves (Bunce 1998, Eamus et al. 2008). The strong limitations imposed by stomatal conductance on carbon assimilation (Cunningham and Read 2003*b*, Peters et al. 2018), especially at higher temperatures, may have been a factor for tropical forest dieback in recent years (Powers et al. 2020), and these are possibly linked to a reduced carbon sink strength in the tropics (McDowell et al. 2018, Hubau et al. 2020, Sullivan et al. 2020, Tagesson et al. 2020).

Another potential constraint in the temperature and VPD responses of photosynthesis is water availability. Drought usually covaries with high temperatures because more water is lost at higher temperatures (covarying with high VPD) than in low VPD conditions. Water availability can impose a large constraint on the temperature response of photosynthesis. Several studies have shown that the temperature optimum of photosynthesis is reduced with lower *g*_s_ (Lin et al. 2012, Kumarathunge et al. 2020), resulting in drought being a large constraint to plant growth. While *M. globosa* did not experience drought stress, low water availability might have been influencing the physiological responses of *E. tereticornis* to warming at EucFACE at the time of measurements. Our study focused on warming responses in the field in a paired design, which, at EucFACE, coincided with high VPD and drought. One limitation of this study is not explicitly taking water potentials into account.

At the temperate EucFACE site, high *T*_air_ during the summer of 2019–20 induced a strong photosynthetic acclimation response with warming. This may have resulted from *T*_leaf_ well beyond the temperature optimum for photosynthesis in this species. However, there was no statistically significant reduction in photosynthetic capacity, *A*_max_, indicating that the reduction in light-saturated *A*_net_ is likely more limited by stomatal conductance in response to higher LAVPD in warmed leaves rather than reduced photosynthetic capacity. Several studies on *Eucalyptus* species have shown reduced rates of the main biochemical delimiters of photosynthetic capacity, *V*_cmax_ and *J*_max_, with warming (Crous et al. 2013, Aspinwall et al. 2016). A similar trend of reduced photosynthetic capacity (either *A*_max_ or the rate of electron transport at CO_2_ saturation, *J*_max_) has been found in tropical species grown in the glasshouse (Cunningham and Read 2003*b*, Scafaro et al. 2017, Crous et al. 2018, Dusenge et al. 2021, Choury et al. 2022). While it is highly likely that low replication and high intraspecific variation may have contributed to a non-significant response of *A*_max_ to warming in our study, most of the studies referenced above occurred in model conditions rather than in situ in the canopy of mature forest trees.

Given our expectation of reduced respiration rates as part of a plant’s strategy to cope with warming and reducing carbon loss, dark respiration rates were similar when measured at a common temperature in warmed and non-warmed leaves in both species. This result suggests that respiration rates did not adjust to warmer leaf temperatures. This non-acclimation response in both species could manifest itself for different reasons. The extreme warm and dry conditions at EucFACE may have resulted in already low respiration rates in reference leaves of *E. tereticornis* (Crous et al. 2011), which did not further reduce with additional warming. However, in the tropics, *Myristica* may not have acclimated respiration (Figure 7), similar to findings by Carter et al. (2021). However, respiration rates measured at a common temperature were lower in *Myristica* compared with the temperate *Eucalyptus*, similar to findings based on much broader datasets of Atkin et al. (2015) and Crous et al. (2022), which reported lower respiration rates at a common temperature in tropical biomes compared with temperate biomes.

### Larger-scale implications and conclusions

The lack of understanding leaf temperature dynamics in natural settings impedes how we model plant functioning in current and future environments. Ambient air temperatures are a poor proxy for leaf temperature in physiological models, especially when accounting for large temporal and spatial differences between them (Michaletz et al. 2016, Rey-Sanchez et al. 2017). Ecosystem and global land-surface vegetation models should simulate Δ*T*_leaf–air_ and benchmark this against measured canopy Δ*T*_leaf–air_ patterns in natural settings (e.g., Figure 5) to avoid erroneous estimations of forest carbon fluxes (Dong et al. 2017, Rey-Sanchez et al. 2017). Moreover, we need to better understand the interplay between high leaf temperatures, VPD, stomatal conductance and plant water status, especially with more extreme temperatures and VPD predicted in the future. Currently, most models predict increased transpiration with higher VPDs (Medlyn et al. 2011, Prentice et al. 2014), whereas field experiments have measured reduced transpiration at very high VPD (Duursma et al. 2008, Drake et al. 2018). Although plant water status greatly affects the ratio of photosynthesis to transpiration in plants, extreme temperatures can decouple the relationship between photosynthesis and transpiration (Ameye et al. 2012, Drake et al. 2018), but it remains to be seen if this decoupling occurs much in natural settings. Moreover, the lack of reduced respiration with warming, if common at large scales, can switch forests from a carbon sink to a source with further warming (Wood et al. 2012).

As a variety of ecosystems may already operate near thermal thresholds for photosynthesis (Doughty 2011, Mau et al. 2018), future warming might lead to heat damage and associated increased risk of canopy dieback, especially in combination with drought (McDowell 2011, Powers et al. 2020). Thus, further field experiments, including warming and drought treatments, are needed to improve our understanding of how higher leaf temperatures and atmospheric drying may affect net CO_2_ assimilation, tree growth and plant climate feedbacks in temperate and tropical forests in future climates (Mercado et al. 2018), including canopy dieback thresholds for various forest types.

## Supplementary Material

crous-etal_leafheatersV6_supplInfo-edited_tpad054

## Data Availability

The data from this manuscript has been published openly in Western Sydney University’s Institutional repository, ResearchDirect with the following doi. https://doi.org/10.26183/9ny4-hd45.
