# Supplementary material for: Similar patterns of leaf temperatures and thermal acclimation to warming in temperate and tropical tree canopies
Source: Tree Physiol. 2023 Apr 26;43(8):1383–99. doi: 10.1093/treephys/tpad054 (PMC10423462; doi:10.1093/treephys/tpad054)

**Supplementary data**

Title: Similar patterns of leaf temperatures and thermal acclimation to warming in temperate and tropical tree canopies.

Crous KY^1^, Cheesman AW^2^, Middleby K^2^, Rogers EIE^1^, Wujeska-Klause A^3^, Bouet AYM^2^, Ellsworth DS^1^, Liddell MJ^2^, Cernusak LA^2^, Barton CVM^1^

^1^ Western Sydney University, Hawkesbury Institute for the Environment, Penrith, NSW Australia

^2^ James Cook University, Centre for Tropical Environmental and Sustainability Science (TESS) and College of Science and Engineering, Cairns, QLD 4878

^3^ Western Sydney University, Urban studies, School of Social Science, Penrith NSW, Australia

The following Supplementary Data are available for this article:

**Supplemental Methods SM1.** Leaf heating experimental details.

**Figure S1**. Photos of the leaf heater design.

**Figure S2**. Thermal infra-red photo (left) of leaf inside an ambient box and outside the box.

**Figure S3**. Long-term soil moisture percentage up to 4.5 m depth at EucFACE between 2013-2022.

**Figure S4**. Range of leaf-to-air vapour pressure deficit (LAVPD) across the air temperatures experienced at EucFACE (panel a) and at DRO (panel b) in reference (ambient in grey) and warmed leaves (warmed in pink).

**Supplemental Methods – Leaf heating experimental details**

The leaf heating experiment involved paired design with reference (e.g., non-heated control) and heated leaves, both supported by similar infrastructure. Each canopy crown had two leaf heater pairs installed in the upper canopy. The clear, plastic boxes (175 mm x 120 mm x 58 mm, made from a recyclable thermoplastic polymer) had holes on top, bottom and sides for air circulation. Leaves were secured in place by fine fishing wire to achieve a consistent distance from the heating wire (Figure S1). For the heated leaves, approximately 80 cm of silicon-coated nichrome heating wire (nickel and chromium, ~40 Ω resistance) was used for heating, providing up to 15 W of heating capacity (corresponding to max. 625 milliamps) with a 24V DC power supply (at 100% duty cycle). The heating wires from the leaf heating boxes were connected to 1.5 mm^2^ 2-core power cables with crimp connectors, and silicon added to make them watertight. The boxes were held in place by small custom-built metal frames, using 3 mm diameter fencing wire, and attached to a large branch via cable ties. The spacing between the reference leaf box and the warmed leaf box was typically about 1 m and installed with similar aspect and light conditions. Thus, per tree, a pair of two 2-core power cables to power the heaters (two per canopy) and one 4-core copper cable to measure the relative leaf temperatures was installed about 1.5 m away from the branch tips, to which the leaf heaters were connected. The cable bundles were tied with either bungee cords or cable ties as close to the main stem as possible and guided down via the main trunk to the ground. At the DRO, the cables were bundled in irrigation tubing (Holman, 19 mm Black Poly Pipe, Bunnings Inc., Australia) to protect cables from tropical rats.

Temperature control was achieved using a CR1000 datalogger (Campbell Scientific), which monitored the temperature difference between heated and reference leaves and adjusted the power to the heater to maintain the desired differential of 4°C. The logger measured the temperature differences every 5 seconds and the power to each heater was updated every 30 seconds with a PID algorithm (i.e., a proportional–integral–derivative controller to employ feedback). Power was regulated by custom-made electronic circuit boards that were installed in series to accommodate eight leaf heater pairs. These circuit boards used MOSFETs to switch the current on and off using pulse width modulation to each of the eight heaters it controlled. The heating time was staggered to minimise surges and cycles through all multiplexer channels modulating the heating via a duty cycle (i.e. how much power a heater received to achieve the desired differential expressed as a percentage).

The difference in temperature between a heated leaf and its reference leaf was measured with a pair of 30-gauge (~0.25 mm diameter) copper-constantan type-T thermocouples (Omega Engineering Inc., Norwalk, CT, USA) connected via a common constantan wire. The copper wires from the paired thermocouples were connected to two connectors of a 4-core cable, the main thermocouple extension wire, via waterproof gel connectors (7.9 mm, insulated, RS Components Pte Ltd Singapore), subsequently connected to an AM25T multiplexer (Campbell Scientific, Logan, UT, USA) and a CR1000 Campbell datalogger.

Fig. S1. Photos of the leaf heater design: The box in metal frame is used to keep the leaf at the same distance from the heated wire at all times (left panel), thermocouple touching the leaf on the abaxial side at all times (upper right panel) and overview of the custom-made circuit boards, AMT25 multiplexer and CR1000 logger (Campbell Scientific, Townsville, Australia) without wire connections in bottom right panel.


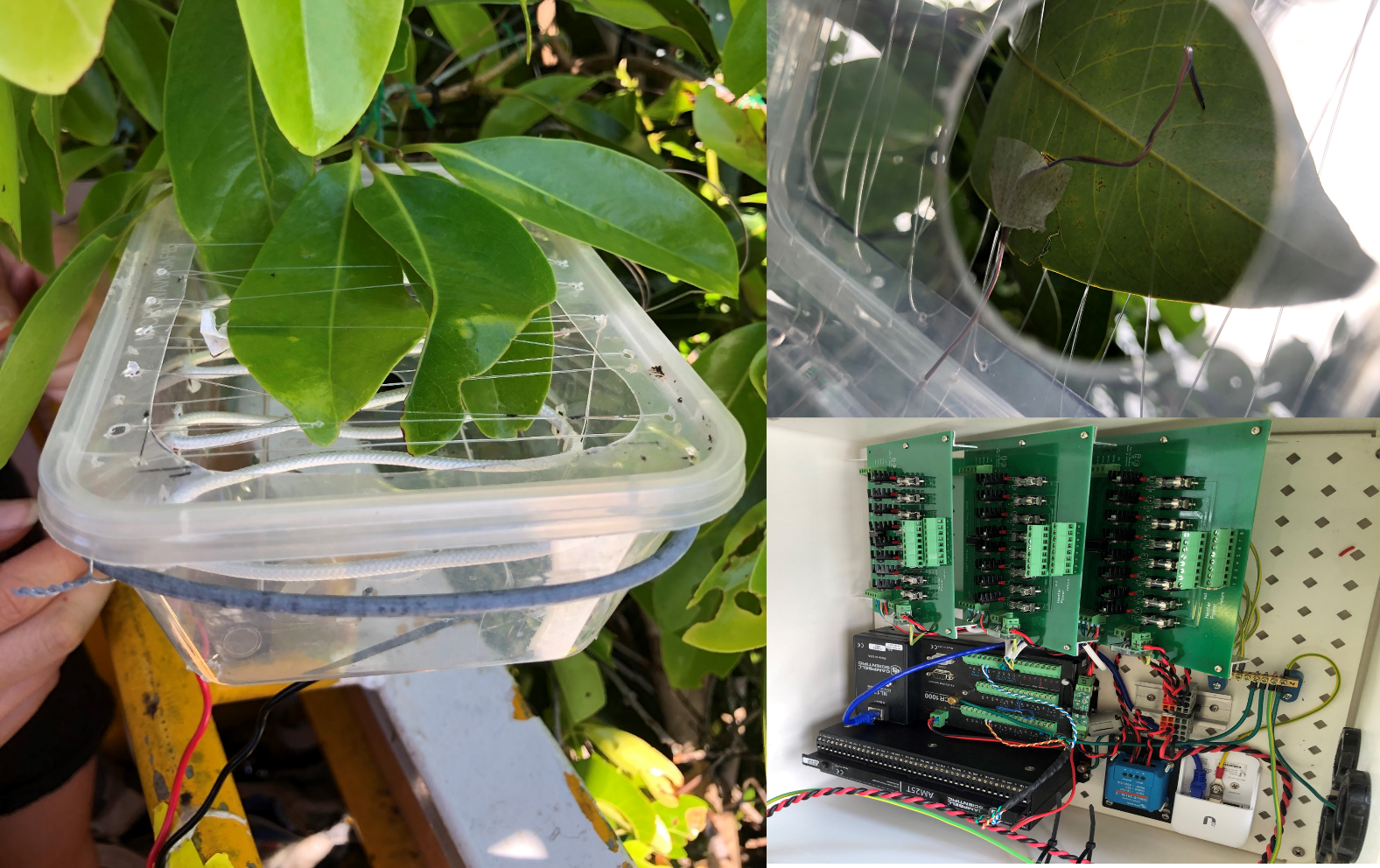


Fig. S2. Thermal infra-red photo (left) of leaf inside an ambient box and outside the box. The colours of the leaves are the same as the background indicating that the box holding the leaf in place does not add to a warmer environment in the canopy (i.e., similar leaf temperatures). Normal photo on the right for comparison.


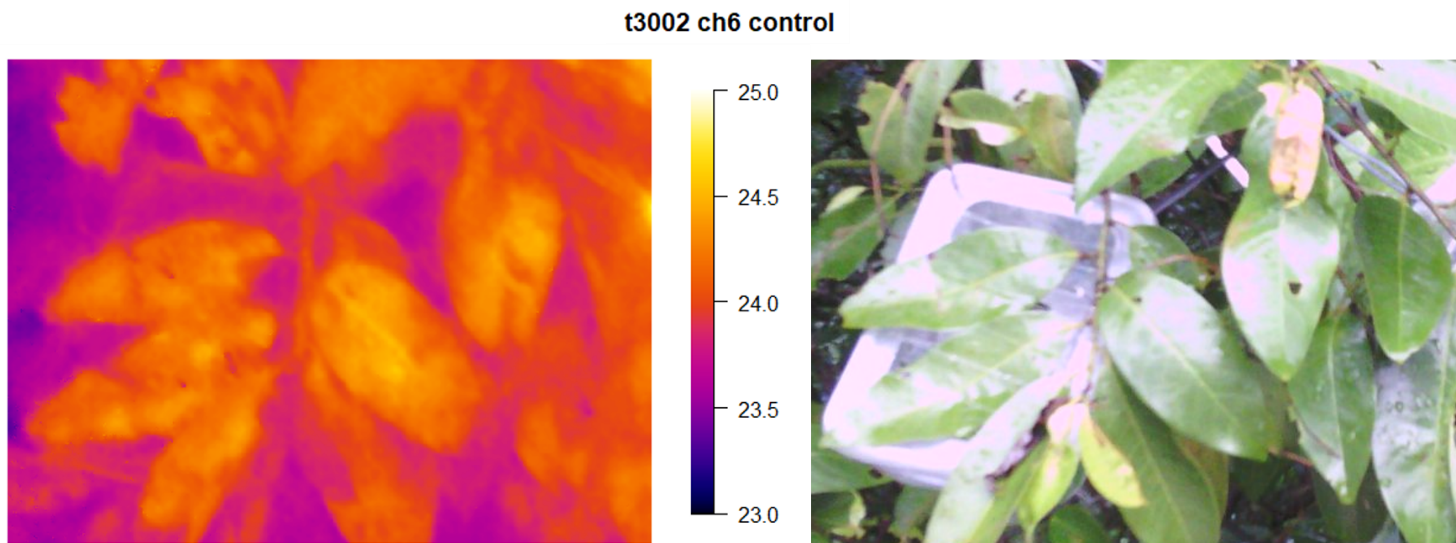


Fig S3. Long-term soil moisture percentage up to 4.5 m depth at EucFACE between 2013-2022 with blue colours indicating wet soils (with 35% soil moisture being saturated for a sandy soil) while green and yellow colours indicate drying. Severe drought starting in 2018 dried the deeper soil moisture levels up until record lows in the summer of 2020, at the time of the experiment (arrow)


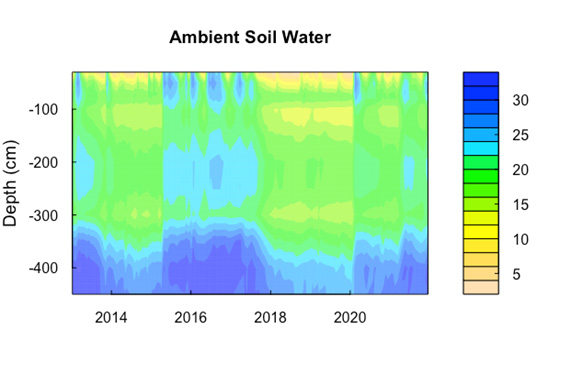


Fig S4. Range of leaf-to-air vapour pressure deficit (LAVPD) across the air temperatures experienced at EucFACE (panel a) and at DRO (panel b) in reference (ambient in grey) and warmed leaves (warmed in pink).


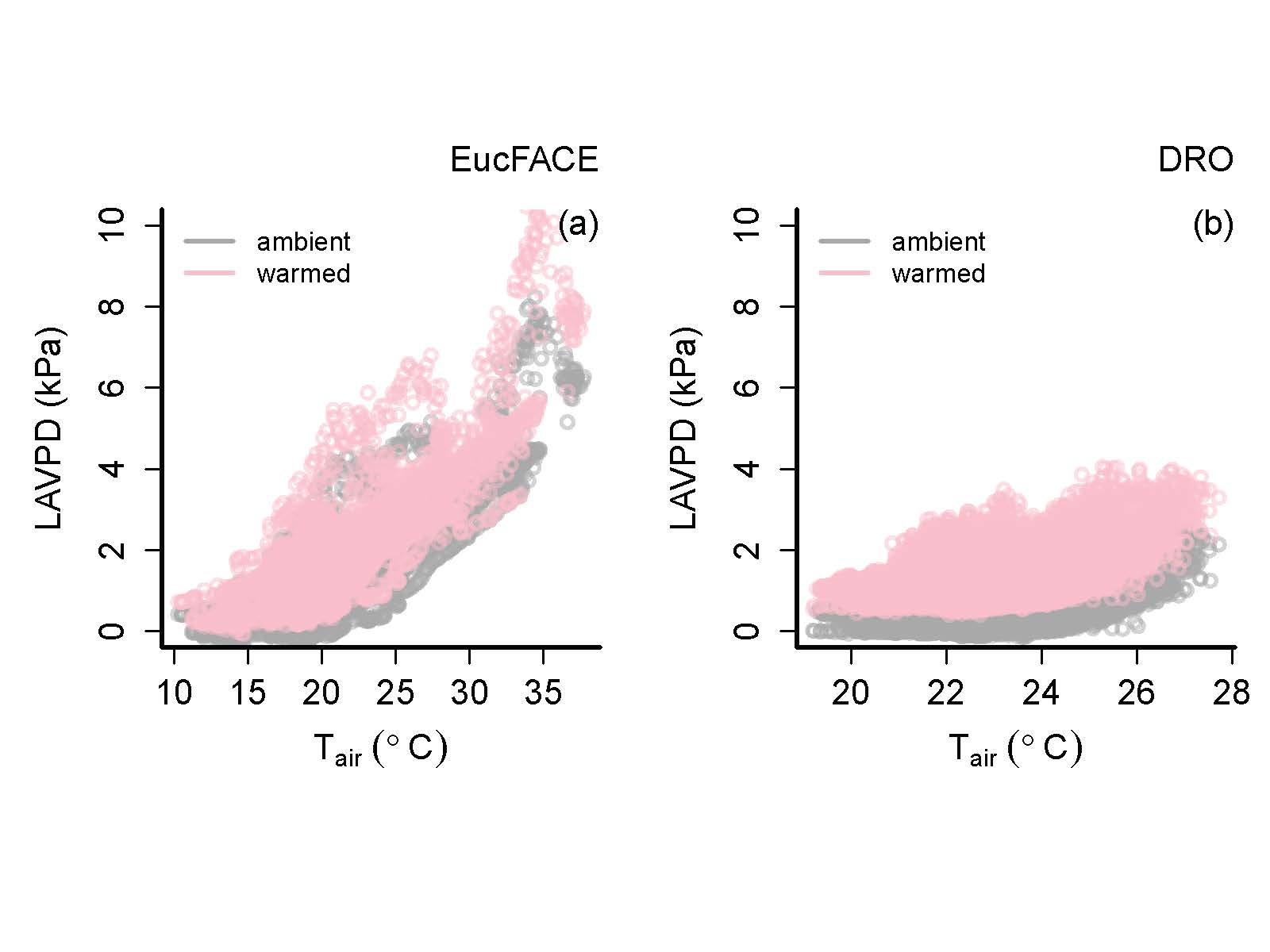

Supplement: crous-etal_leafheatersV6_supplInfo-edited_tpad054 [file crous-etal_leafheatersv6_supplinfo-edited_tpad054.zip › crous-etal_leafheatersV6_supplInfo-edited_tpad054.docx]
